# Evaluation of the marginal and internal fit of endocrown restorations fabricated by the CAD/CAM system using additive manufacturing technique

**DOI:** 10.1007/s00784-026-06891-y

**Published:** 2026-04-29

**Authors:** Eric Arnold Dos Santos Brito, Murilo Montanari Souza, Marianna Soares Nogueira Borges, Maria Julia Lopes Ramos, Julia Godoi-Lopes, Leandro Cardoso, Manoel Damião Sousa-Neto, Jardel Francisco Mazzi Chaves, Camila Tirapelli

**Affiliations:** 1 Departamento de Materiais Dentários e Prótese, Faculdade de Odontologia de Ribeirão Preto, Universidade de São Paulo, Avenida do Café, sn. Bairro Monte Alegre. CEP 14040904, Ribeirão Preto, São Paulo Brazil; 2 Department of Restorative Dentistry, Ribeirão Preto School of Dentistry, University of São Paulo (USP), Ribeirão Preto, São Paulo Brazil

**Keywords:** Endocrown, Marginal and internal fit, Digital workflow, Additive manufacturing

## Abstract

**Objective:**

This study aimed to compare the marginal and internal fit of endocrowns with cavity depths of 3 mm and 5 mm, using two printable resins fabricated by additive manufacturing within a digital workflow.

**Materials and methods:**

Thirty-two human mandibular molars were prepared for endocrowns with cavity depths of 3–5 mm and allocated into four groups (*n* = 8). Two printable resins (Biocrown Diamond and Voxel Print) were evaluated at both preparation depths. The restorations were designed using CAD software and manufactured by DLP-based additive manufacturing. Marginal and internal fit were assessed using micro-computed tomography (micro-CT) from standardized sagittal and coronal sections at predefined anatomical regions. Data were analyzed using two-way repeated-measures ANOVA followed by Tukey’s test (α = 0.05).

**Results:**

Material and preparation depth influenced the marginal and internal fit of endocrown restorations, with a significant interaction with the anatomical region. The highest discrepancies were observed in the axio-occlusal angle, particularly for the 5-mm preparation groups. Significant differences between materials occurred at the axio-occlusal angle for 3-mm preparations and in the marginal gap for 5-mm preparations. No significant differences were detected in the pulpal regions.

**Conclusion:**

Endocrowns showed clinically acceptable marginal adaptation at both preparation depths. However, internal adaptation was region-dependent, with greater discrepancies in the axio-occlusal region. Both resins demonstrated acceptable behavior, although localized differences were observed according to preparation depth and anatomical region.

**Clinical significance:**

Preparation depth and printable resin type may influence the internal adaptation of additively manufactured endocrowns and should be considered during treatment planning.

## Introduction

Endocrown restorations represent a rehabilitative option for endodontically treated teeth, relying on micromechanical and macromechanical retention within the pulp chamber, axial walls, and cavity margins. Their indication includes cases of extensive coronal tissue loss, limited interocclusal space, or curved and narrow root canals [1]. The advancement of digital dentistry, combined with improvements in adhesive approaches, has enabled the development of more precise and conservative restorations. Within this context, marginal and internal fit constitute fundamental parameters for assessing clinical success [2]. Marginal and internal fit have been defined as the vertical distance between the preparation finish line and the cervical margin, and the distance from the internal surface of the restoration to the axial wall of the preparation, respectively [3]. The presence of marginal gaps promotes biofilm accumulation, microleakage, secondary caries, gingival inflammation, and cement exposure, which may ultimately lead to biomechanical failures [4]. Although there is no current consensus regarding the maximum clinically acceptable discrepancy, values ranging from 50 μm to 120 μm have been reported in the literature [5]. Additionally, internal gaps greater than 70 μm may compromise fracture resistance [6].

The advancement of CAD/CAM systems has provided tools capable of producing endocrowns with high precision, improved quality, and reduced treatment time [7, 8]. Despite inherent limitations related to acquisition devices and techniques, several studies have reported favorable outcomes compared with conventional methods, considering the limitations of impression materials and operator-related errors [5, 9]. The digital workflow involves multiple factors that directly influence restoration outcomes and clinical success, including preparation depth, scanner type, scanning technique, software, operator skill [5], as well as restorative material, manufacturing protocol, and cementation technique [9]. Among these, preparation depth is considered a critical factor in the dimensional accuracy of endocrown restorations. The literature indicates that increased depth may compromise digital capture quality by creating shadowed regions, leading to loss of information during geometric reconstruction and favoring misfit, particularly in deeper internal areas [6]. One study [10] reported that preparations of 3 to 4 mm exhibited greater internal discrepancies compared with 2 mm depths, especially at the pulpal floor. These findings were attributed to the geometric complexity of deeper regions, combined with optical limitations of intraoral scanners and the 3D reconstruction process, which may result in excessive smooth internal angles. Similarly, another study [11] found increased misfit with preparation depths of 4 mm compared with 2 mm. Overall, these findings suggest that preparation depth is a determining factor for internal fit, particularly in critical axial and pulpal regions.

Within the digital workflow, two main manufacturing approaches are recognized: subtractive manufacturing (SM), performed by milling, and additive manufacturing (AM), which has gained prominence due to advantages such as reduced material waste, high reproducibility of complex geometries, and the ability to produce multiple restorations simultaneously [12]. In AM, printing parameters, including build orientation, layer thickness, curing time, and light intensity, directly influence the physical properties and dimensional accuracy of restorations. Layer thicknesses of approximately 50 μm and printing angles between 45° and 60° have been associated with improved marginal and internal fit [13–15]. The absence of cutting forces in AM preserves marginal geometry and eliminates milling-induced defects; however, inherent limitations remain. Dimensional accuracy may vary depending on the technology and parameters used, with internal discrepancies being particularly affected by Z-axis resolution, build orientation, sharp internal angles, and post-curing polymerization effects, especially in deeper preparations [16]. Despite these limitations, previous studies have reported favorable marginal and internal fit outcomes for additively manufactured restorations compared with subtractive methods [17–19]. Additionally, restorative material characteristics influence adaptation. 3D-printed resins exhibit lower elastic modulus and flexural strength (approximately 80–135 MPa), which may favor marginal continuity, whereas ceramic materials used in SM, despite superior mechanical strength, are more susceptible to milling-related defects and dimensional inconsistencies [16, 20, 21].

Although additive manufacturing appears promising, there is still insufficient evidence regarding endocrowns produced digitally with varying preparation depths. Thus, it is necessary to investigate the impact of additive manufacturing on the marginal and internal fit of endocrown restorations with different cavity depths. Accordingly, this study aimed to compare the internal and marginal fit of endocrown restorations fabricated using additive manufacturing (DLP) at different preparation depths. The null hypothesis was that, for endocrowns fabricated using a digital workflow, no differences in marginal or internal adaptation would be observed among restorations produced with different 3D-printing resins and preparation depths.

## Materials and methods

This study was approved by the Ethics Committee of the School of Dentistry of Ribeirão Preto, University of São Paulo (CAAE: 75268023.7.0000.5419). The sample size was determined through an a priori power analysis using G*Power software (Heinrich-Heine-Universität Düsseldorf, Germany). The effect size was estimated based on marginal adaptation data reported by Xiao et al. [22]. Considering α = 0.05 and 80% statistical power for the two-way ANOVA design, the minimum required sample size was calculated as seven specimens per group. Thus, eight specimens were included in each group. The groups analyzed were: Biocrown Diamond; Voxel print; endocrown preparation depth (3 mm), and endocrown preparation depth (5 mm). A total of 32 samples (human mandibular first molars) were obtained from the Tooth Bank of the School of Dentistry of Ribeirão Preto, University of São Paulo. Endocrown restorations were fabricated for natural teeth. Inclusion criteria consisted of sound mandibular molars, absence of fractures, and complete root formation. Teeth meeting one or more of the following conditions were excluded: endodontically treated teeth and extensively damaged crowns.

### Endodontic protocol

After sample selection, all specimens underwent standardized endodontic treatment. Conventional access opening was performed using a spherical diamond bur (801 L, Jota AG, Rüthi, Switzerland), followed by an Endo Z bur (Beavers Jet Burs, Morrisburg, Canada), both operated in high speed (Dabi Atlante, Ribeirão Preto, SP, Brazil) under continuous water irrigation (Ingle, 1976). Each canal was initially explored with a #10 K-file (Dentsply Maillefer, Ballaigues, Switzerland) until apical patency was reached. Working length (WL) was established by subtracting 1 mm from this measurement, using the respective root’s buccal cusp as reference. Biomechanical preparation was carried out using a reciprocating technique with the Reciproc 50.05 instrument (VDW GmbH, Munich, Germany). The instrument was operated with a 6:1 reduction contra-angle (Sirona SN 25185; VDW GmbH, Munich, Germany), coupled to the SMR 114,058 micromotor (VDW GmbH, Munich, Germany) connected to the VDW Silver electric motor (VDW GmbH, Munich, Germany). Torque and speed parameters were those pre-set for the Reciproc system according to manufacturer’s instructions. Instrumentation was performed passively using short in-and-out pecking motions. After every three pecks, the instrument was removed and cleaned with gauze. This cycle was repeated until the WL was reached, after which the instrument was used with short circumferential strokes, applying light apical pressure to contact all canal walls. At each instrument removal, irrigation was performed with 2.5% sodium hypochlorite, followed by aspiration and reinsertion of irrigant with a disposable plastic syringe (Ultradent Products., South Jordan, UT, USA) and NaviTip needle (Ultradent Products, South Jordan, UT, USA). Final irrigation consisted of 2 mL of 17% EDTA for 5 min, followed by 5 mL of 2.5% sodium hypochlorite. Canals were dried with R50 paper points (Reciproc, VDW GmbH, Munich, Germany) and obturated using the single-cone technique with R50 gutta-percha cones (Reciproc, VDW GmbH, Munich, Germany). AH Plus sealer was manipulated according to the manufacturer’s instructions. Sealer was carried into the canal with a K-file, and the gutta-percha cone coated with sealer was inserted with circular motions until reaching the WL. Excess material at the canal orifice was removed with a heated Paiva condenser (Golgran Indústria e Comércio de Instrumentos Odontológicos, São Caetano do Sul, SP, Brazil). While the gutta-percha remained plastic, vertical condensation was performed with a cold condenser, applying gentle apical pressure. After obturation, specimens were individually stored in numbered Eppendorf tubes and kept in an incubator at 37 °C and 100% humidity for 14 days to allow complete sealer setting.

### Sample preparation

After the endodontic treatment, the direct restorative procedures on the pulpal wall of the cavity were performed as follows: etching with 37% phosphoric acid (Vivadent Ivoclar AG, Schaan, Liechtenstein) for 30 s, rinsing with a water spray for 30 s, drying with an air jet, application of Ybond Universal adhesive (Yller, Pelotas, Brazil) using a microbrush, followed by light curing for 20 s with an LED curing unit (Valo, Ultradent) at an intensity of 1200 mW/cm². The pulpal floor and canal openings were filled with a low-viscosity composite resin (Tetric N-Flow Bulk Fill, Ivoclar Vivadent, Schaan, Liechtenstein) and light-cured using the same curing unit (Valo, Ultradent) to obtain a symmetrical surface parallel to the occlusal plane, allowing adjustments in preparation depth [23, 24].

The samples were then randomly assigned to two groups according to the preparation depth, measured from the buccal axial wall to the chamber floor, approximately 3.0–5.0 mm. To create a 1-mm-wide chamfer finish line (2 mm above the cemento-enamel junction) and prepare the axial walls, diamond burs #4138 and #4138FF (American Burrs, Palhoça, Santa Catarina, Brazil) were used, maintaining rounded internal angles and 8° of divergence in the pulpal chamber. Subsequently, an occlusal reduction of 2 mm was performed using a tapered diamond bur #3131 (American Burrs, Palhoça, Santa Catarina, Brazil) under high-speed rotation with irrigation, and measurements were confirmed with a Williams periodontal probe (Golgran, São Caetano do Sul, São Paulo, Brazil) [25, 26].

### Sample digitization

The samples were positioned in a lower-jaw dental mannequin with artificial teeth (MOM Manequins Odontológicos, Marília, São Paulo, Brazil), in the positions corresponding to teeth 36 and 46, using the cemento-enamel junction as a reference to standardize digitization by enabling clear separation between tooth and gingiva. The teeth were fixed to the mannequin with condensation silicone (Zetalabor, Zhermack, Rovigo, Italy) applied internally. Digitization was performed by a single previously calibrated operator in a controlled environment. Each specimen was scanned using the PrimeScan intraoral scanner (Dentsply Sirona, North Carolina, United States), following the linear scanning technique proposed [27].

### Virtual planning

The acquired data were imported into the InLab system (Sirona Dental Systems, Bensheim, Germany), and endocrown restorations were designed according to the desired specifications. The CAD parameters used for the restorations were: Radial spacer (80 μm); Occlusal spacer (80 μm); Proximal contact strength (25 μm); Occlusal contact strength (25 μm); Dynamic contact strength (25 μm); Minimum radial thickness (2000 μm); Minimum occlusal thickness (2000 μm); Margin thickness (50 μm); Ramp width (50 μm); Ramp angle (60°).

### Manufacturing of the restorations

A total of 32 endocrown restorations were fabricated using a DLP-based 3D printer (Flashforge The Hunter, California, USA). The restorations were distributed according to the experimental groups: Group 1 – Biocrown Diamond resin, 3-mm depth (*n* = 8); Group 2 – Biocrown Diamond resin, 5-mm depth (*n* = 8); Group 3 – Voxel Print resin, 3-mm depth (*n* = 8); and Group 4 – Voxel Print resin, 5-mm depth (*n* = 8). The printable resins used contained silanized filler within an inorganic matrix [28, 29]. Printing parameters were established according to the manufacturer’s recommendations for each material. For Biocrown Diamond, the following settings were used: layer thickness: 50 μm, curing time: 5.0 s, adhesion-layer curing time: 20.0 s, transition layers: 8, light intensity: 100%. For Voxel print, the parameters were: layer thickness: 100 μm, curing time: 4.2 s, adhesion-layer curing time: 35.0 s, transition layers: 8, light intensity: 100%. After printing, the restorations were sprayed with isopropyl alcohol and cleaned with a microbrush to remove excess resin. Post-curing was performed using UV light for 30 min (Voxel Print) and 20 min (Biocrown Diamond).

### Cementation of the restorations

The internal surface treatment of each restoration was performed according to the manufacturer’s recommendations for each material. For the 3D-printed resins, the cementation surface was sandblasted with aluminum oxide (25–50 μm), followed by suction-assisted air removal. A silane coupling agent (Silane, Ultradent, South Jordan, Utah, United States.) was applied for 60 s and gently air-dried for 5 s. A dual-cure composite-based luting agent (SetPP – SDI, Bayswater, Victoria, Austrália ) was then applied to the internal surfaces of the prosthetic restoration. Light curing was performed for 20 s on each surface using an LED curing unit (Valo, Ultradent, South Jordan, Utah, United States.) at 1200 mW/cm². After excess material was removed, the restoration margins were covered with an oxygen-inhibiting gel, as instructed by the manufacturer.

### Evaluation of marginal and internal adaptation

After cementation, the samples were scanned using a high-resolution micro-CT scanner SkyScan 1174 v.2 (Bruker microCT, Kontich, Belgium), set according to the manufacturer’s specifications. Before scanning, the buccal surface of each specimen was marked with a permanent marker. To prevent movement during scanning, each specimen was individually embedded in utility wax attached to a metallic micropositioner, which was firmly secured to the turntable via a manual control screw inside the micro-CT chamber. In this position, each specimen remained perpendicular to the radiation source, with the crown facing upward, reducing the risk of image distortion. The following parameters were used: 50 kVp, 800 µA, isotropic resolution of 14.6 μm, 360° rotation around the vertical axis, a total of 1 frame, rotation step of 0.5°, and a 0.5-mm aluminum filter. After scanning, the two-dimensional projections were reconstructed using NRecon v.1.7.4.2 (Bruker microCT), applying ring artifact correction (value 5), beam-hardening correction (35%), smoothing (value 7), and contrast histogram limits ranging from 0.02 to 0.25. The reconstructed images were saved in Tagged Image File Format (TIFF). At the end of scanning, the specimens were immersed again in saline solution and stored in an incubator (37 °C, 95% relative humidity).

### 3D Reconstruction of the images

This step consisted of reconstructing the axial sections from the angular projection images using the modified Feldkamp cone-beam reconstruction algorithm in NRecon v.1.7.3.1 (Bruker microCT, Kontich, Belgium), in order to fully represent the internal microstructure of each sample. Ring artifact reduction was applied with a value of 5 (scale 0–20), beam-hardening correction with 35% (scale 0–100%), smoothing with a value of 7 (scale 0–10), and a contrast histogram range from 0.02 to 0.25. The reconstructed axial sections were saved in Bitmap (BMP) format.

### Image processing and analysis

The reconstructed images obtained with NRecon (Bruker microCT) were processed in DataViewer v.1.5.1.2 for three-dimensional alignment and generation of standardized slices. Image volumes were loaded and viewed in 3D mode (“Load for 3D viewing”), enabling precise adjustment of the specimen in the axial, coronal and sagittal planes. Positioning ensured that the long axis of the root canal remained parallel to the sagittal plane and perpendicular to the axial plane, thereby preserving geometric alignment among specimens. After achieving appropriate spatial alignment, reference lines were centered on the root canal to standardize the slice location across all specimens. For quantitative analysis, multiple standardized sections were exported: two sagittal and two coronal sections per specimen, all passing through the canal central axis; these sections were equally spaced to capture circumferential and longitudinal variation. Exported images intended for measurement were saved in bitmap (.bmp) format.

**Fig. 1:**
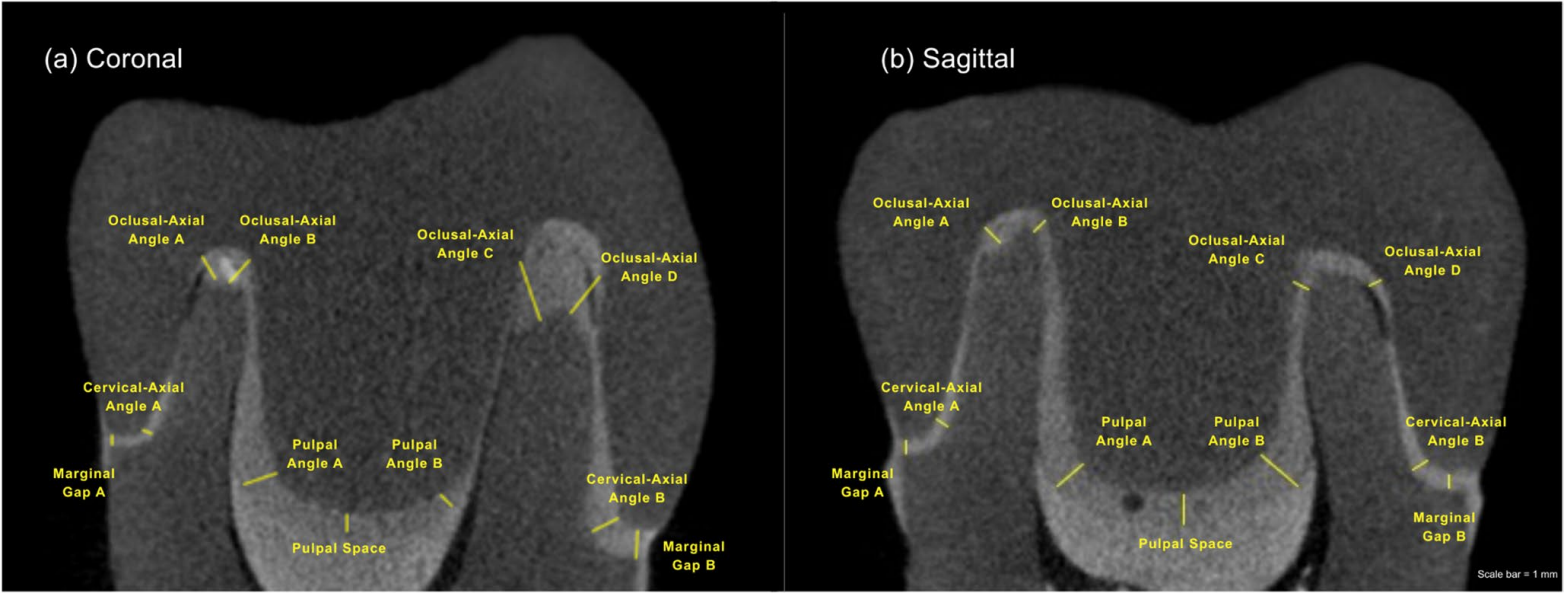
Representative micro-CT sections illustrating the anatomical measurement locations used to evaluate marginal and internal fit. The predefined points include marginal gap (A and B), cervical-axial angle (A and B), axio-occlusal angle (A–D), pulpal angle (A and B), and pulpal space. The (**a**) coronal and (**b**) sagittal images shown correspond to different sectional views of the same specimen and are presented to illustrate the measurement locations. For the quantitative analysis, each specimen was evaluated using four standardized sections (two sagittal and two coronal), with measurements performed at the same predefined locations in all sections

For quantitative analysis, four standardized micro-CT sections were evaluated for each specimen: two sagittal and two coronal sections, all passing through the central axis of the root canal. These sections were equally spaced to capture circumferential and longitudinal variation along the restoration–tooth interface. In each section, cement line thickness was measured using ImageJ software (version 1.54; National Institutes of Health, Bethesda, MD, USA) at 11 predefined anatomical points along the restoration–tooth interface, following the measurement protocol described by Pilecco et al. [21]. The evaluated points included: Marginal Gap A and B; Cervical–Axial Angle A and B; Axial–Occlusal Angle A, B, C, and D; Pulpal Angle A and B; and Pulpal Space, as shown in Fig. 1. All measurements were performed by two trained operators to ensure methodological consistency. Intra-examiner reliability was assessed by the intraclass correlation coefficient (ICC = 95%) and demonstrated excellent agreement.

Thus, each specimen generated 11 measurements per section, resulting in a total of 44 measurements per specimen (11 measurement points × 4 sections). Measurements obtained from the four sections were averaged for each anatomical point, resulting in 11 representative values per specimen. Subsequently, these points were grouped according to anatomical regions to facilitate interpretation and statistical analysis. The marginal region corresponded to the mean of Marginal Gap A and B; the cervical–axial region corresponded to the mean of Cervical–Axial Angle A and B; the axio-occlusal region corresponded to the mean of Axial–Occlusal Angle A–D; and the pulpal angle region corresponded to the mean of Pulpal Angle A and B. The pulpal space corresponded to a single measurement point and consequently was not averaged. These final regional values (marginal gap, cervical–axial angle, axio-occlusal angle, pulpal angle, and pulpal space) were used for statistical analysis, as demonstrated in Table 1.

**Table 1:** Detailing the 11 micro-CT measurement points* into the anatomical regions used for statistical analysis. For each specimen, measurements were obtained from four standardized sections (two sagittal and two coronal), and the mean value of the sections was calculated for each measurement point. The resulting 11 values were then grouped by anatomical region to obtain representative measurements for marginal gap, cervical–axial angle, axio-occlusal angle, and pulpal angle, while pulpal space corresponded to a single measurement point

Measurement points (micro-CT)	Region used for analysis
Marginal Gap A + Marginal Gap B	Marginal gap
Cervical-Axial Angle A + Cervical-Axial Angle B	Cervical-axial angle
Axial-Occlusal Angle A + B + C + D	Axio-occlusal angle
Pulpal Angle A + Pulpal Angle B	Pulpal angle
Pulpal Space	Pulpal space

*Figure 1 illustrates the measurement points

Three-dimensional volumetric segmentation of the cement layer was considered but not employed due to technical limitations that could introduce systematic error. Specifically, gray-scale overlap between the luting cement and the additive resin restoration produced ambiguous threshold boundaries and pronounced partial-volume effects, rendering automated or semi-automated segmentation unreliable without extensive manual correction. Manual segmentation in this context is highly operator-dependent and would increase subjectivity and risk of bias. The use of contrast agents or dyes to improve segmentation was not feasible within the study protocol. For this reason, a standardized two-dimensional approach employing multiple sections (multiple sagittal and coronal sections with multiple calibrated measurement points) was chosen; this method reduces sampling bias, captures circumferential variability, and provides robust, reproducible thickness data across specimens.

### Statistical analysis

For each specimen (*n* = 32), 11 measurement points obtained from micro-CT analysis were first calculated as the mean of the measurements performed in four standardized sections (two sagittal and two coronal). These measurement points were subsequently grouped according to anatomical regions to obtain representative values for marginal gap, cervical–axial angle, axio-occlusal angle, pulpal angle, and pulpal space (Table 1). Marginal and internal fit data were then analyzed considering resin material (Biocrown Diamond and Voxel Print) and cavity depth (3 mm and 5 mm) as between-subject factors, and anatomical region (marginal, cervical-axial, axio-occlusal, pulpal angle, and pulpal space) as a within-subject factor. A two-way repeated-measures analysis of variance (RM ANOVA) was performed to evaluate the main and interaction effects on fit values. When sphericity assumptions were violated, Greenhouse–Geisser correction was applied. Tukey’s multiple comparisons test was used for post hoc pairwise comparisons. Partial eta-squared (ηp²) was calculated as a measure of effect size. The level of significance was set at *P* < 0.05. Statistical analyses were performed using GraphPad Prism software (version 8.0.1; GraphPad Software, San Diego, CA, USA).

## Results

**Fig. 2:**
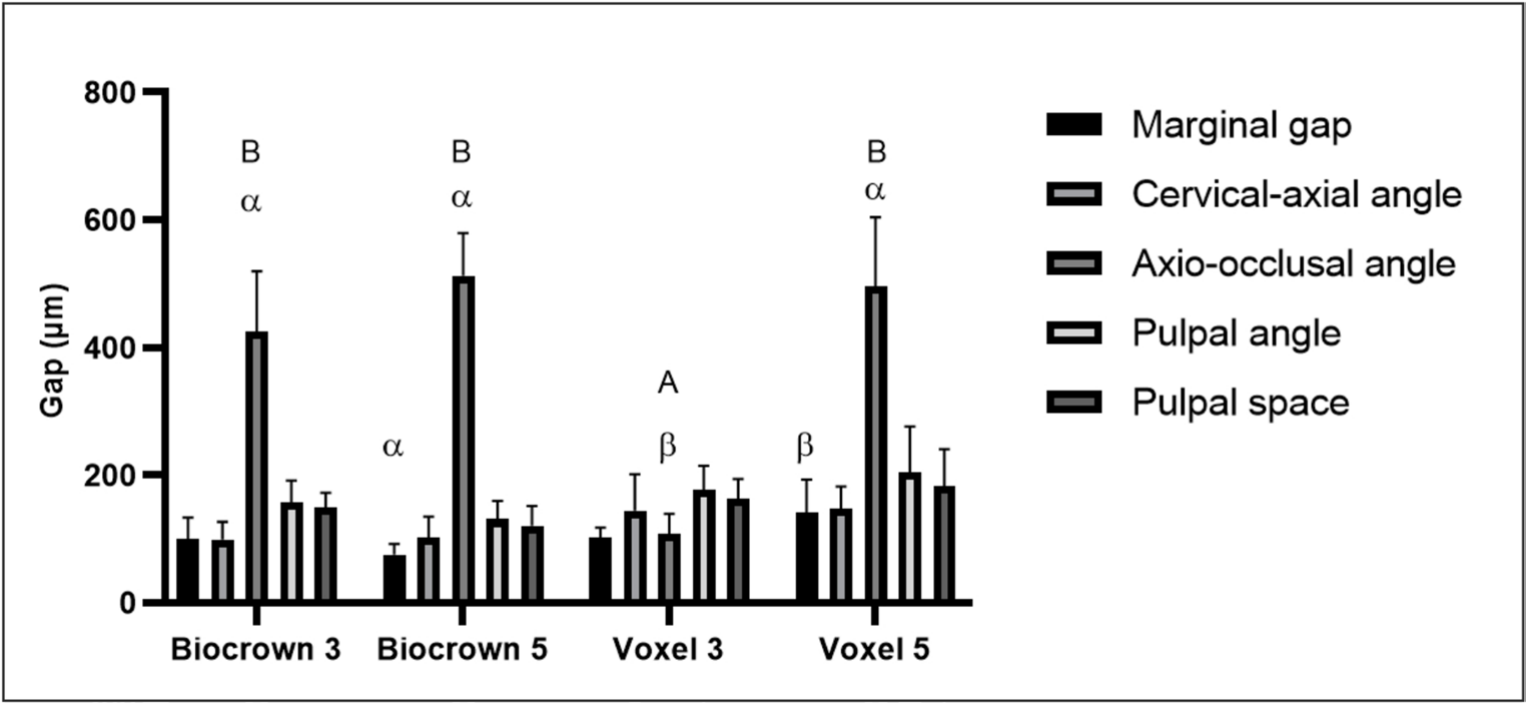
Graphical representation of the measurements considering resins (Biocrown and Voxel) and preparation depths (3 mm and 5 mm). Different uppercase letters indicate statistically significant differences for the same resin. Different Greek letters indicate statistically significant differences between resins for the same preparation depth (*P* < 0.0001)

**Fig. 3:**
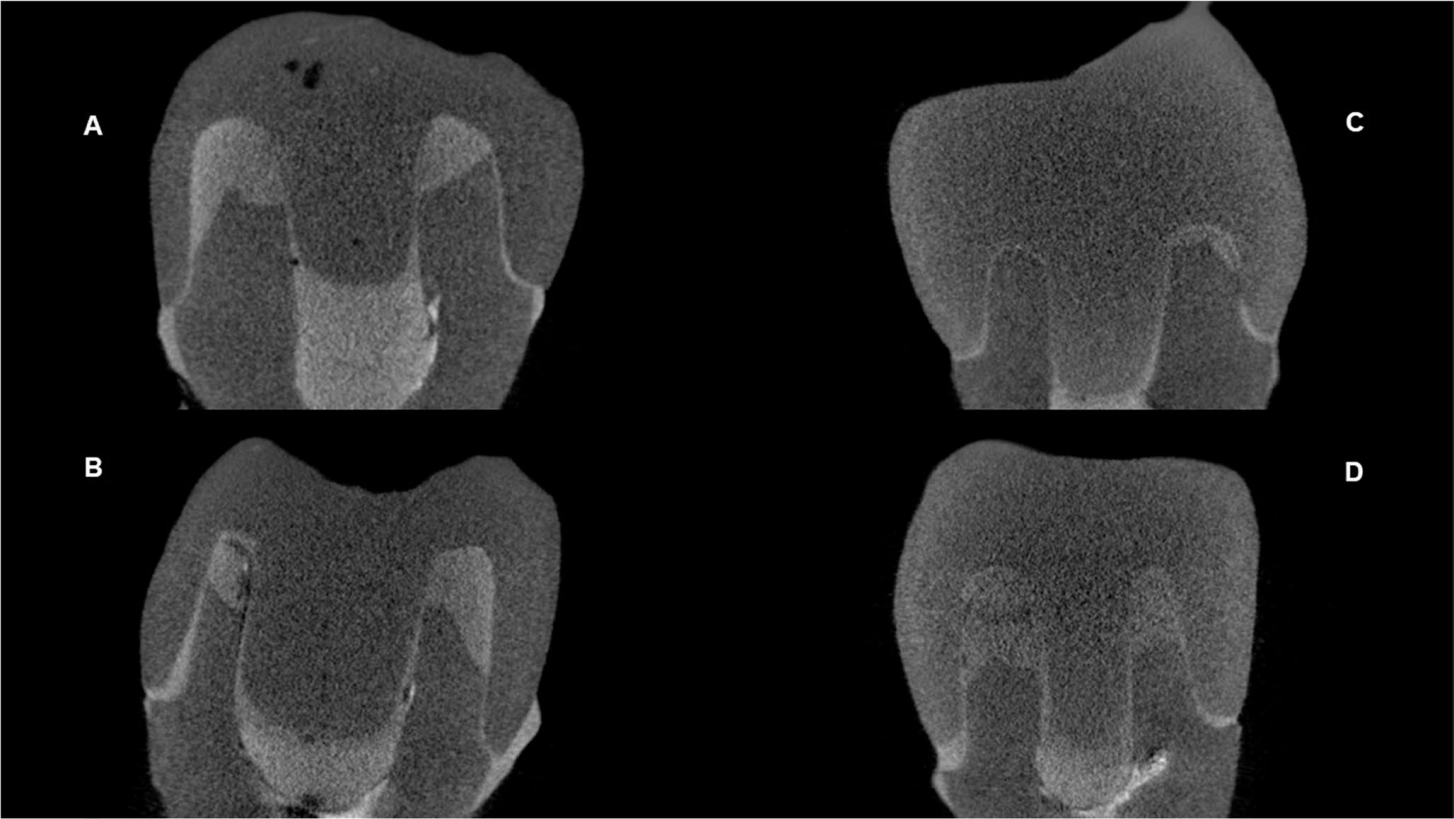
Representative micro-CT coronal sections of the experimental groups: Biocrown 3 mm (**A**), Biocrown 5 mm (**B**), Voxel 3 mm (**C**), and Voxel 5 mm (**D**). These images illustrate one of the standardized sections used for quantitative analysis. For each specimen, four sections were evaluated (two sagittal and two coronal), all passing through the central axis of the root canal

The results are presented in Figs. 2 and 3; Tables 2, 3 and 4. Figure 2 illustrates the distribution of marginal and internal fit values according to resin material and preparation depth, while Table 3 summarizes the mean and standard deviation values for all evaluated regions. Table 4 presents the complete two-way repeated-measures ANOVA results, including interaction effects and effect sizes (partial η²), and Table 3 provides Tukey-adjusted pairwise comparisons with corresponding 95% confidence intervals. Figure 3 shows representative reconstructed coronal micro-CT sections of each experimental group.

**Table 2:** Mean and standard deviation (SD) of measurement values (µm) for each group

		Marginal	Cervical-axial angle	Axio-occlusal angle	Pulpal angle	Pulpal space
Biocrown 3 mm	Mean	100.413	99.291	425.923	156.904	149.667
	SD	33.048	27.615	93.434	34.573	22.897
Biocrown 5 mm	Mean	75.712	101.864	512.463	130.844	121.333
	SD	16.769	33.215	67.034	28.577	30.520
Voxel 3 mm	Mean	101.625	144.176	108.433	176.684	163.725
	SD	16.287	57.778	30.914	38.219	30.925
Voxel 5 mm	Mean	140.816	147.705	496.129	204.894	182.378
	SD	52.488	34.362	108.503	71.461	58.900

**Table 3:** Two-way repeated-measures ANOVA results evaluating the effects of group (Biocrown 3 mm, Biocrown 5 mm, Voxel 3 mm, Voxel 5 mm) and anatomical region (marginal, cervical-axial, axio-occlusal, pulpal angle, pulpal space) on marginal and internal fit values. F-values, degrees of freedom (df), p-values, and partial eta-squared (ηp²) are presented. Greenhouse–Geisser correction was applied when sphericity assumptions were violated

Source	df (DFn, DFd)	F	*p*-value	Partial η²
Group	(3, 28)	9.818	0.0001	0.496
Region (GG corrected)	(1.525, 42.70)	250.9	< 0.0001	0.900
Group × Region	(12, 112)	37.59	< 0.0001	0.801

Greenhouse–Geisser ε = 0.3813

**Table 4:** Tukey-adjusted pairwise comparisons (simple effects within each region) showing mean differences, 95% confidence intervals (CI), and adjusted p-values (α = 0.05)

Marginal gap			
Comparison	Mean Diff (µm)	95% CI (µm)	Adj. *p*
Biocrown 3 vs. Biocrown 5	24.70	−15.12 to 64.52	0.2912
Biocrown 3 vs. Voxel 3	−1.212	−40.92 to 38.49	0.9997
Biocrown 3 vs. Voxel 5	−40.40	−105.7 to 24.87	0.3025
Biocrown 5 vs. Voxel 3	−25.91	−49.94 to − 1.887	0.0328*
Biocrown 5 vs. Voxel 5	−65.10	−126.8 to − 3.420	0.0388*
Voxel 3 vs. Voxel 5	−39.19	−100.8 to 22.46	0.2556

Cervical–axial angle			
Comparison	Mean Diff (µm)	95% CI (µm)	Adj. p
Biocrown 3 vs. Biocrown 5	−2.573	−47.15 to 42.00	0.9982
Biocrown 3 vs. Voxel 3	−44.88	−114.1 to 24.33	0.2565
Biocrown 3 vs. Voxel 5	−48.41	−93.98 to − 2.846	0.0359*
Biocrown 5 vs. Voxel 3	−42.31	−113.0 to 28.42	0.3247
Biocrown 5 vs. Voxel 5	−45.84	−94.96 to 3.277	0.0711
Voxel 3 vs. Voxel 5	−3.530	−74.65 to 67.59	0.9988

Axio–occlusal angle			
Comparison	Mean Diff (µm)	95% CI (µm)	Adj. p
Biocrown 3 vs. Biocrown 5	−86.54	−206.3 to 33.18	0.1963
Biocrown 3 vs. Voxel 3	317.5	207.6 to 427.4	< 0.0001*
Biocrown 3 vs. Voxel 5	−70.21	−217.8 to 77.35	0.5275
Biocrown 5 vs. Voxel 3	404.0	324.0 to 484.1	< 0.0001*
Biocrown 5 vs. Voxel 5	16.33	−118.1 to 150.8	0.9829
Voxel 3 vs. Voxel 5	−387.7	−515.0 to − 260.4	< 0.0001*

Pulpal angle			
Comparison	Mean Diff (µm)	95% CI (µm)	Adj. p
Biocrown 3 vs. Biocrown 5	26.06	−20.24 to 72.36	0.3888
Biocrown 3 vs. Voxel 3	−19.78	−72.81 to 33.25	0.7037
Biocrown 3 vs. Voxel 5	−47.99	−133.7 to 37.71	0.3675
Biocrown 5 vs. Voxel 3	−45.84	−95.38 to 3.700	0.0736
Biocrown 5 vs. Voxel 5	−74.05	−158.7 to 10.55	0.0900
Voxel 3 vs. Voxel 5	−28.21	−114.8 to 58.41	0.7610

Pulpal space			
Comparison	Mean Diff (µm)	95% CI (µm)	Adj. p
Biocrown 3 vs. Biocrown 5	28.33	−11.27 to 67.93	0.2039
Biocrown 3 vs. Voxel 3	−14.06	−54.03 to 25.91	0.7336
Biocrown 3 vs. Voxel 5	−32.71	−102.4 to 36.93	0.4947
Biocrown 5 vs. Voxel 3	−42.39	−87.04 to 2.258	0.0653
Biocrown 5 vs. Voxel 5	−61.04	−132.2 to 10.09	0.0999
Voxel 3 vs. Voxel 5	−18.65	−89.89 to 52.59	0.8561

* Significant at α = 0.05 (Tukey-adjusted)

The two-way repeated-measures ANOVA revealed a significant interaction between group and anatomical region (F(12,112) = 37.59, *p* < 0.0001, ηp² = 0.801), indicating that the influence of material and preparation depth on fit values depended on the anatomical region evaluated (Table 4). Significant main effects were also observed for group (F(3,28) = 9.818, *p* = 0.0001, ηp² = 0.496) and region (F(1.525,42.70) = 250.9, *p* < 0.0001, ηp² = 0.900; Greenhouse–Geisser corrected). Overall, the axio-occlusal angle demonstrated the highest discrepancy values in most groups (Fig. 2), whereas the cervical-axial, pulpal angle, and pulpal space regions showed comparatively lower discrepancy values.

When comparing materials at the same preparation depth, a statistically significant difference was observed at the axio-occlusal angle between Biocrown 3 mm and Voxel 3 mm (*p* < 0.0001). At the 5-mm preparation depth, a significant difference between materials was detected for the marginal gap, with Voxel 5 mm showing higher discrepancy values than Biocrown 5 mm (*p* = 0.0388).

When comparing preparation depths within the same resin, a statistically significant difference was observed only for the Voxel resin at the axio-occlusal angle, with higher discrepancy values at 5 mm compared with 3 mm (*p* < 0.0001). No significant depth-related differences were detected for the Biocrown resin in any region. No statistically significant differences were observed at the pulpal angle or pulpal space. All pairwise comparisons were Tukey-adjusted, and corresponding 95% confidence intervals are presented in Table 3.

## Discussion

The present study evaluated the influence of preparation depth and restorative material on the marginal and internal fit of additively manufactured endocrown restorations. The null hypothesis was rejected, as both preparation depth and restorative material influenced the marginal and internal fit of endocrown restorations.

Marginal discrepancies ranged approximately from 80 to 145 μm among the experimental groups, remaining within clinically acceptable limits reported in the literature. Classical investigations have suggested that marginal gaps of approximately 120 μm are clinically acceptable for indirect restorations, with some studies reporting acceptable values up to 150 μm depending on clinical conditions. This indicates that the marginal discrepancies observed in the present study fall within ranges considered compatible with long-term clinical performance [30, 31].

On the contrary, internal adaptation, mainly at axio-occlusal angle, exceeded clinically acceptable values, being possibly influenced by an interaction between preparation depth, material characteristics, and anatomical region. The Voxel resin demonstrated a significant increase in discrepancy when comparing the 3 mm and 5 mm preparations (from 108 μm to 496 μm) at axio-occlusal, indicating that deeper cavities amplify internal inaccuracies for this material. This dependent behavior between depth and material suggests that the manufacturing limitations of the material become more pronounced as geometric complexity and depth increase. In this context, it is important to discuss printing parameters as in this study, both materials were printed according to manufacturer recommendations using a 100 μm and 50 μm layer thickness for Voxel and Biocrowns, respectively. Layer thickness directly governs Z-axis resolution and the ability to reproduce internal geometries. Thicker layers can increase discretization error and the staircase effect, particularly in regions with complex curvature such as the axio-occlusal angle [32, 33]. These limitations can become more pronounced in deeper preparations, where cumulative layering error and reduced light penetration during polymerization may further compromise dimensional accuracy. This interaction between preparation depth and manufacturing resolution may explain the pronounced discrepancies observed in the Voxel 5 mm group, compared to Voxel 3. In literature, reduced accuracy and inferior mechanical performance is associated with greater compared with thinner layers, as reported by Borella et al. (2023) and Farkas et al. (2023). Additionally, the layer-by-layer fabrication process may introduce cumulative inaccuracies and interlayer inconsistencies, further compromising the reproduction of sharp internal angles such as the axio-occlusal transition, as previously reported in studies evaluating the influence of printing parameters and build strategies on dimensional accuracy [14, 15, 18].

Nevertheless, the present results suggest that layer thickness alone does not fully explain internal discrepancies, since Biocrown, fabricated with thinner layers (50 μm), also exhibited considerable misfit. This findings reinforces that internal adaptation can be multifactorially governed by the interaction between material properties, manufacturing parameters, and geometric complexity. Interestingly, Biocrown did not exhibit significant differences between 3 mm and 5 mm preparations; consistently showing the highest discrepancy values at the axio-occlusal region across groups. Possibly, for this material, internal adaptation is less influenced by the dependence between layer thickness and preparation depth and factors such as material composition, polymerization behavior, and dimensional stability, particularly in regions of abrupt geometric transition can be playing a role. Unfortunately, the material compositions of the materials studied are not fully detailed by the manufacturers and scientific studies are not available covering such points, but it is possible to observe that Voxel Print contains zirconia-based inorganic fillers, whereas Biocrown Diamond is primarily composed of glass-based particles, also the amount of inorganic fillers varies between resins, being greater for Voxel. Previous studies have demonstrated that the type and distribution of inorganic fillers in photocurable resins can significantly influence their polymerization behavior, mechanical properties, and dimensional stability [34]. The Voxel 3 mm group presented comparatively low discrepancy values in the axio-occlusal region, within clinically acceptable ranges, indicating that under less complex geometric conditions, this material is capable of achieving satisfactory internal adaptation.

Regarding the differences between marginal and internal discrepancies, mainly for the axio-occlusal region, this finding may be discussed by the critical transition between axial and occlusal surfaces; this region concentrates abrupt geometric changes that challenged the accuracy of layer-by-layer fabrication. Similar behavior has been reported by Suksuphan et al. [18], who observed that 3D-printed restorations may achieve acceptable marginal adaptation while still presenting limitations in internal fit, particularly in regions with greater geometric complexity or thickness. In this context, factors related to image acquisition, CAD design smoothing, printing parameters, polymerization shrinkage and minimal internal adjustments before cementation can be connected with such a discrepancy.

From a clinical perspective, internal discrepancies approaching 500 μm represent a substantial deviation from commonly accepted thresholds, and may compromise the biomechanical integrity of the restorations. Greater internal discrepancies may lead to increased cement thickness, which has been associated with reduced mechanical stability at the restoration–tooth interface and increased stress concentration under functional loading [35, 36]. In addition, increased cement volume may amplify polymerization shrinkage stress, potentially compromising interfacial integrity and contributing to gap formation or debonding over time [37, 38]. From a biomechanical perspective, this condition may favor crack initiation and propagation, consequently reducing fracture resistance [39, 38]. These effects may be further exacerbated in additively manufactured restorations, in which the layer-by-layer fabrication process and potential interfacial defects may act as structural discontinuities [32, 40, 38]. Furthermore, deeper intracoronal preparations, although not necessarily increasing fracture load, may alter stress distribution within the remaining tooth structure, increasing the risk of catastrophic failure patterns [41, 42, 43].

This study has limitations such as this in vitro design, which does not fully reproduce clinical conditions. Although micro-computed tomography enables full three-dimensional evaluation, the present analysis relied on standardized two-dimensional sections. This approach was adopted due to the technical challenges associated with volumetric segmentation of cement spaces in additively manufactured resin restorations, particularly due to grayscale overlap between materials. In addition, only a single set of printing and post-curing parameters was used, as different manufacturing conditions—such as printing parameters and temperature—were not evaluated and may influence the internal adaptation of the restorations. Furthermore, clinical cementation procedures may introduce additional variables that affect internal fit. Future research should compare different additive and subtractive workflows under simulated clinical conditions, investigate additional printable materials, and further optimize manufacturing parameters to fabricated endocrowns.

Although marginal adaptation was within clinically acceptable limits, the high internal discrepancies observed in the axio-occlusal region exceed commonly accepted thresholds and should not be interpreted as clinically acceptable internal adaptation. These discrepancies, particularly those approaching 500 μm, may have relevant biomechanical implications and compromise the structural performance of the restoration.

## Conclusions

The endocrown restorations demonstrated acceptable marginal performance at both evaluated preparation depths. However, internal discrepancies were pronounced in the axio-occlusal region, particularly in deeper preparations, exceeding accepted thresholds.

## Data Availability

The data that support the findings of this study are available from the corresponding author upon reasonable request.

## References

[1] Shams A, Elsherbini M, Elsherbiny AA, Özcan M, Sakrana AA (2022) Rehabilitation of severely destructed endodontically treated premolar teeth with a novel endocrown system: biomechanical behavior assessment through 3D finite element and in vitro analyses. J Mech Behav Biomed Mater 126:105031. 10.1016/j.jmbbm.2021.105031 34922296 10.1016/j.jmbbm.2021.105031

[2] Topkara C, Keleş A (2022) Examining the adaptation of modified endocrowns prepared with CAD-CAM in maxillary and mandibular molars: A microcomputed tomography study. J Prosthet Dent 127:744–749. 10.1016/j.prosdent.2020.12.003 33454117 10.1016/j.prosdent.2020.12.003

[3] Holmes JR, Bayne SC, Holland GA, Sulik WD (1989) Considerations in measurement of marginal fit. J Prosthet Dent 62:405–408. 10.1016/0022-3913(89)90170-4 2685240 10.1016/0022-3913(89)90170-4

[4] Sanches IB, Metzker TC, Kappler R, Oliveira MV, Carvalho AO, Castor Xisto Lima EM (2023) Marginal adaptation of CAD-CAM and heat-pressed lithium disilicate crowns: A systematic review and meta-analysis. J Prosthet Dent 129:34–39. 10.1016/j.prosdent.2021.03.021 34147239 10.1016/j.prosdent.2021.03.021

[5] Ayres G, Parize H, Mendonça LM, Kubata BR, Tirapelli C (2025) Is the digital workflow more efficient for manufacturing partial-coverage restorations? A systematic review. J Prosthet Dent 133:1438–1447. 10.1016/j.prosdent.2023.08.005 37716898 10.1016/j.prosdent.2023.08.005

[6] Hasanzade M, Sahebi M, Zarrati S, Payaminia L, Alikhasi M (2021) Comparative evaluation of the internal and marginal adaptations of CAD/CAM endocrowns and crowns fabricated from three different materials. Int J Prosthodont 34:341–347. 10.11607/ijp.6389 31856266 10.11607/ijp.6389

[7] de Paula Silveira AC, Chaves SB, Hilgert LA, Ribeiro AP (2017) Marginal and internal fit of CAD-CAM-fabricated composite resin and ceramic crowns scanned by two intraoral cameras. J Prosthet Dent 117:386–392. 10.1016/j.prosdent.2016.07.017 27677214 10.1016/j.prosdent.2016.07.017

[8] AlHelal AA (2024) Biomechanical behavior of all-ceramic endocrowns fabricated using CAD/CAM: A systematic review. J Prosthodont Res 68:50–62. 10.2186/jpr.JPR_D_22_00296 37286503 10.2186/jpr.JPR_D_22_00296

[9] Abduljawad DE, Rayyan MR (2022) Marginal and internal fit of lithium disilicate endocrowns fabricated using conventional, digital, and combination techniques. J Esthet Restor Dent 34:707–714. 10.1111/jerd.12902 35294099 10.1111/jerd.12902

[10] Gaintantzopoulou MD, El-Damanhoury HM (2016) Effect of preparation depth on the marginal and internal adaptation of computer-aided design/computer-assisted manufacture endocrowns. Oper Dent 41:607–616. 10.2341/15-146-L 27379835 10.2341/15-146-L

[11] Shin Y, Park S, Park JW, Kim KM, Park YB, Roh BD (2017) Evaluation of the marginal and internal discrepancies of CAD-CAM endocrowns with different cavity depths: An in vitro study. J Prosthet Dent 117:109–115. 10.1016/j.prosdent.2016.03.025 27460311 10.1016/j.prosdent.2016.03.025

[12] Al-Dulaijan YA, Aldamanhori R, Algaoud H et al (2024) Internal and marginal fits of 3D-printed provisional prostheses: Comparative effect of different printing parameters. Front Oral Health 5:1491984. 10.3389/froh.2024.1491984 39726768 10.3389/froh.2024.1491984 PMC11669682

[13] Abdelhady W, Abozaid D, Mohammed M, Ashraf M, Metwally M, Mohammed H (2025) A systematic review on influence of printing layer thickness on the marginal and internal fit of 3D-printed fixed dental prostheses. Odontology. 10.1007/s10266-025-01241-y 10.1007/s10266-025-01241-y PMC13320041 41264112

[14] Yang MS, Kim SK, Heo SJ, Koak JY, Park JM (2022) Investigation of the marginal fit of a 3D-printed three-unit resin prosthesis with different build orientations and layer thicknesses. J Adv Prosthodont 14:250–261. 10.4047/jap.2022.14.4.250 36105878 10.4047/jap.2022.14.4.250 PMC9444483

[15] Jang G, Kim SK, Heo SJ, Koak JY (2024) Fit analysis of stereolithography-manufactured three-unit resin prosthesis with different 3D-printing build orientations and layer thicknesses. J Prosthet Dent 131:301–312. 10.1016/j.prosdent.2021.11.031 36653209 10.1016/j.prosdent.2021.11.031

[16] Revilla-León M, Meyers MJ, Zandinejad A, Özcan M (2019) A review on chemical composition, mechanical properties, and manufacturing workflow of additively manufactured current polymers for interim dental restorations. J Esthet Restor Dent 31:51–57. 10.1111/jerd.12438 30367716 10.1111/jerd.12438

[17] Abdulkareem MA, Al-Shamma AMW (2024) Marginal adaptation and fracture resistance of 3D-printed and CAD/CAM-milled definitive resin matrix ceramic crowns. Int J Comput Dent 27:355–363. 10.3290/j.ijcd.b4494301 37823542 10.3290/j.ijcd.b4494301

[18] Suksuphan P, Krajangta N, Didron PP, Wasanapiarnpong T, Rakmanee T (2024) Marginal adaptation and fracture resistance of milled and 3D-printed CAD/CAM hybrid dental crown materials with various occlusal thicknesses. J Prosthodont Res 68:326–335. 10.2186/jpr.JPR_D_23_00089 37438119 10.2186/jpr.JPR_D_23_00089

[19] Cantó-Navés O, Michels K, Figueras-Alvarez O, Fernández-Villar S, Cabratosa-Termes J, Roig M (2023) In vitro comparison of internal and marginal adaptation between printed and milled onlays. Mater (Basel) 16:6962. 10.3390/ma16216962 10.3390/ma16216962 PMC10650727 37959559

[20] Magne P, Carvalho AO, Bruzi G, Giannini M (2015) Fatigue resistance of ultrathin CAD/CAM complete crowns with a simplified cementation process. J Prosthet Dent 114:574–579. 10.1016/j.prosdent.2015.04.014 26119017 10.1016/j.prosdent.2015.04.014

[21] Pilecco RO, da Rosa LS, Baldi A et al (2024) How do different intraoral scanners and milling machines affect the fit and fatigue behavior of lithium disilicate and resin composite endocrowns? J Mech Behav Biomed Mater 155:106557. 10.1016/j.jmbbm.2024.106557 38657286 10.1016/j.jmbbm.2024.106557

[22] Xiao P, Zheng Z, Zhang Y, Zeng Y, Yan W (2024) Accuracy and adaptation of one-piece endodontic crowns fabricated through 3D printing and milling. J Prosthet Dent 132:422–433. 10.1016/j.prosdent.2024.05.01 38880678 10.1016/j.prosdent.2024.05.011

[23] El Ghoul WA, Özcan M, Ounsi H, Tohme H, Salameh Z (2020) Effect of different CAD-CAM materials on the marginal and internal adaptation of endocrown restorations: An in vitro study. J Prosthet Dent 123:128–134. 10.1016/j.prosdent.2018.10.024 31027958 10.1016/j.prosdent.2018.10.024

[24] Hayes A, Duvall N, Wajdowicz M, Roberts H (2017) Effect of endocrown pulp chamber extension depth on molar fracture resistance. Oper Dent 42:327–334. 10.2341/16-097-L 28467258 10.2341/16-097-L

[25] Nassar H (2022) Internal fit and marginal adaptation of CAD-CAM lithium disilicate endocrowns fabricated with conventional impression and digital scanning protocols: An in vitro study. Egypt Dent J 68:3793–3808. 10.21608/edj.2022.162266.2256

[26] Taha D, Spintzyk S, Schille C et al (2018) Fracture resistance and failure modes of polymer infiltrated ceramic endocrown restorations with variations in margin design and occlusal thickness. J Prosthodont Res 62:293–297. 10.1016/j.jpor.2017.11.003 29241944 10.1016/j.jpor.2017.11.003

[27] Salem MM, Elmahy WA, Nasr DM (2024) Effect of different intraoral scanning strategies on the marginal and internal fit of CAD-CAM inlay restorations: An in vitro study. J Prosthet Dent 131:518e1–518e9. 10.1016/j.prosdent.2023.11.007 10.1016/j.prosdent.2023.11.007 38040555

[28] FGM Dental Group (2024) Voxelprint – FGM Dental Group. https://fgmdentalgroup.com/intl/voxelprint/. Accessed 9 Dec 2025

[29] Makertech Labs (2013) –) About Us – Makertech Labs. https://makertechlabs.com/. Accessed 9 Dec 2025

[30] McLean JW, von Fraunhofer JA (1971) The estimation of cement film thickness by an in vivo technique. Br Dent J 131:107–111. 10.1038/sj.bdj.4802708 5283545 10.1038/sj.bdj.4802708

[31] Papadiochou S, Pissiotis AL (2018) Marginal adaptation and CAD-CAM technology: A systematic review of restorative material and fabrication techniques. J Prosthet Dent 119:545–551. 10.1016/j.prosdent.2017.07.001 28967399 10.1016/j.prosdent.2017.07.001

[32] Borella PS, Alvares LAS, Ribeiro MTH et al (2023) Physical and mechanical properties of four 3D-printed resins at two different thick layers: An in vitro comparative study. Dent Mater 39(8):686 37357046 10.1016/j.dental.2023.06.002

[33] Farkas AZ, Galatanu SV, Nagib R (2023) The Influence of Printing Layer Thickness and Orientation on the Mechanical Properties of DLP 3D-Printed Dental Resin. Polym (Basel) 15(5):1113 Published 2023 Feb 23. 10.3390/polym15051113 10.3390/polym15051113 PMC10007339 36904351

[34] Karntiang P, Ikeda H, Nagamatsu Y, Shimizu H (2025) Photocurable resin composites with silica micro- and nano-fillers for 3D printing of dental restorative materials. J Compos Sci 9:405. 10.3390/jcs9080405

[35] Al Fodeh RS, Al-Johi OS, Alibrahim AN et al (2023) Fracture strength of endocrown maxillary restorations using different preparation designs and materials. J Mech Behav Biomed Mater 148:106184. 10.1016/j.jmbbm.2023.106184 37839334 10.1016/j.jmbbm.2023.106184

[36] Bahadır HS, Bulut AC, Yılmaz B (2024) Effect of preparation design on the fracture resistance and fracture patterns of 3D printed one-piece endodontic crowns. J Prosthet Dent 132(3):592e1–59592. 10.1016/j.prosdent.2023.05.015. e7 10.1016/j.prosdent.2024.06.004 38960755

[37] Nandalur KR, Alshehri AH, Chourasia HR et al (2025) Impact of margin type and material choice on stress distribution in endocrown restorations: A 3D finite element study. Med Sci Monit 31:e948308. 10.12659/MSM.948308 40383938 10.12659/MSM.948308 PMC12101097

[38] Abad-Coronel C, Durán Urdiales D, Benalcázar Arias MV et al (2025) *Flexural strength, fatigue behavior, and microhardness of three-dimensional printed resin material for indirect restorations: A systematic review.* Materials. (Basel) 18(3):556. 10.3390/ma18030556 10.3390/ma18030556 PMC11818428 39942222

[39] Karntiang P, Ikeda H, Nagamatsu Y, Shimizu H (2025) Photocurable resin composites with silica micro- and nano-fillers for 3D printing of dental restorative materials. J Compos Sci 9(8):405

[40] Flottes Y, Smail Y, Palomino-Durand C, Attal JP, Ceinos R, François P, Dursun E. (2026) Properties of 3D printed resins for definitive dental restorations: A systematic review. J Prosthet Dent 135(4):e6-e27. 10.1016/j.prosdent.2025.11.040 10.1016/j.prosdent.2025.11.040 41419355

[41] Donmez MB, Ersöz E, Çakmak G, Diken Türksayar AA, Altinci P, Yilmaz B (2026) Fracture strength of additively manufactured one-piece endodontic crowns in resins for definitive use: Effect of material type, margin configuration, and pulp chamber depth. J Prosthet Dent 135(4):806.e1-806.e8. 10.1016/j.prosdent.2025.11.027 10.1016/j.prosdent.2025.11.027 41372040

[42] Gaafar SS, El Ballouli D, Rayyan M et al (2025) Fracture resistance, failure mode and restorability of CAD/CAM zirconia endocrowns with different pulpal extension depths bonded to maxillary molars: an in vitro study. BMC Oral Health 25(1):176 39893449 10.1186/s12903-025-05466-9 PMC11786583

[43] 42 Abuabboud O, Marinescu AG, Paven M et al (2025) Influence of cement thickness, dentine thickness, and intracoronal depth on the fracture resistance of 3D-printed endocrowns: a pilot in vitro study. Dentistry J 13(6):263 10.3390/dj13060263 PMC12191686 40559166

